# The Effects of Pharmacological and Non-Pharmacological Interventions on Symptom Management and Quality of Life among Breast Cancer Survivors Undergoing Adjuvant Endocrine Therapy: A Systematic Review

**DOI:** 10.3390/ijerph17082950

**Published:** 2020-04-24

**Authors:** Carmen W.H. Chan, Daria Tai, Stephanie Kwong, Ka Ming Chow, Dorothy N.S. Chan, Bernard M.H. Law

**Affiliations:** 1 The Nethersole School of Nursing, Faculty of Medicine, The Chinese University of Hong Kong, Hong Kong, China; whchan@cuhk.edu.hk (C.W.H.C.); kmchow@cuhk.edu.hk (K.M.C.); dorothycns@cuhk.edu.hk (D.N.S.C.); 2 Department of Psychology, The University of British Columbia, Vancouver, BC V6T 1Z4, Canada; daria.tai@alumni.ubc.ca; 3 Department of Pharmacology and Toxicology, University of Toronto, Toronto, ON M5S 1A8, Canada; swykwong200@gmail.com

**Keywords:** Breast cancer, endocrine therapy, hormonal therapy, intervention, quality of life, symptom, survivorship care

## Abstract

Breast cancer survivors need to undergo adjuvant endocrine therapy after completion of curative treatments to prevent disease recurrence. These individuals often experience symptoms which are detrimental to their quality of life (QOL). Implementation of interventions for effective symptom management among these survivors is warranted. This review provides an overview of studies on the effectiveness of the previously developed interventions for breast cancer survivors undergoing adjuvant endocrine therapy on symptom alleviation and enhancement of QOL or health-related QOL (HRQOL). Five electronic databases were employed in the literature search. Study selection, data extraction and critical appraisal of the included studies were conducted by three authors independently. Twenty-four studies were included. Both pharmacological and non-pharmacological interventions are effective in addressing the symptoms associated with adjuvant endocrine therapy among the breast cancer survivors, and in improving their QOL, although discrepancies were noted between the studies in terms of the significance of these effects. Pharmacological and non-pharmacological interventions can be effective for symptom management among breast cancer survivors. Their implementation is recommended for effective survivorship care for these individuals. Further research on intervention development for breast cancer survivors is recommended to provide further evidence for the utility of the explored interventions in survivorship care for these patients.

## 1. Introduction

Breast cancer patients, who have completed primary treatments such as surgery, chemotherapy and radiotherapy, require additional therapies for preventing recurrence of the disease. Adjuvant endocrine therapies, which encompass hormonal or endocrine therapy using drugs such as tamoxifen and aromatase inhibitors (AI), are known to serve this purpose [1,2,3,4,5]. These drugs are prescribed to breast cancer patients after the completion of primary treatment, which need to be taken daily for a duration of five years for the therapy to be effective [6]. Premature discontinuation of adjuvant endocrine therapy was reported to be prevalent among cancer survivors [1,7]. This highlights that patient non-adherence to adjuvant therapies is a major issue. 

Previous studies demonstrated that patient non-adherence to adjuvant endocrine therapy is primarily caused by the adverse side effects on patients’ quality of life (QOL) that are associated with such therapy [8,9]. These common side effects may include hot flashes, night sweats, fatigue, sleep disturbance, sexual dysfunction, joint stiffness, joint dysfunction and joint pain [9,10,11,12,13]. These symptoms may also negatively affect patients’ functional ability. For example, muscle pain caused by aromatase inhibitor-based therapies could lead to physical impairment [14]. Moreover, adverse effects associated with adjuvant endocrine therapy were demonstrated to lead to patients’ early discontinuation of such adjuvant treatment [15]. 

Non-adherence to and/or early discontinuation of adjuvant endocrine therapy would exhibit negative effects on the well-being of breast cancer survivors. It could lead to an increase in breast cancer mortality and an increased risk of cancer recurrence [1,16]. This is compounded with higher costs incurred for cancer treatment and poorer QOL [16,17]. The development of effective strategies for the management of side effects associated with adjuvant endocrine therapy is therefore of paramount importance. Over the past years, there has been an increasing number of published studies reporting the development of interventions which would be effective in addressing the aforementioned adjuvant endocrine therapy-induced symptoms. Given the potential of these interventions in improving the well-being of breast cancer survivors, conduction of a systematic review that examines the findings of these interventional studies and summarizes the major components of these interventions is worthwhile.

Previously, several reviews have been published, focusing on the various types of interventions that target specific symptoms associated with adjuvant endocrine therapy [18,19,20,21,22]. To the best of our knowledge, a systematic review that examines the effectiveness of the different types of interventions on various known adjuvant endocrine therapy-induced symptoms, overall QOL and functional ability of patients is yet to be conducted. The aim of the present review is to provide an overview on the findings of randomized control trials reporting the effectiveness of various types of interventions targeted for breast cancer survivors undergoing adjuvant endocrine therapy on the improvement of their QOL or health-related QOL (HRQOL) and functional ability. The review also examines the effect of these interventions on alleviating common symptoms reported to be caused by adjuvant endocrine therapy, including hot flashes, night sweats, fatigue, sleep disturbance, sexual dysfunction, joint stiffness, joint dysfunction and joint pain. Findings of this review may provide strategic directions to intervention development for breast cancer patients who are in high need for effective management of the aforementioned undesirable side effects of adjuvant endocrine therapy and reduce their non-adherence to the therapy. 

## 2. Methods

### 2.1. Search Strategy

We performed a comprehensive literature search in November 2019 to identify articles reporting interventions or programs developed for breast cancer survivors undergoing adjuvant endocrine therapy, from date of inception to November 2019. This enabled a comprehensive review of the articles that report the effectiveness of the relevant interventions published previously. Five databases, including PubMed, OVID MEDLINE (since 1946), EMBASE (since 1910), PsycINFO (since 1806), and CINAHL Complete, were used for the search. We employed the search strategy that is set out in Table 1. 

### 2.2. Inclusion and Exclusion Criteria

To be included in the review, research articles should describe interventions developed for breast cancer survivors who are: (1) not undergoing curative treatments such as chemotherapy and radiotherapy; and (2) on adjuvant endocrine therapy. Included studies should report interventions that aim to improve one or more of the following outcomes: (1) endocrine symptoms (vaginal dryness, night sweats and hot flashes); (2) sexual symptoms; (3) joint symptoms (joint pain and stiffness); (4) fatigue; (5) sleep problems; and (6) QOL or HRQOL. Only studies with a randomized control trial (RCT) design and those written in English are included. Studies that included samples not entirely comprising participants undergoing adjuvant endocrine therapy at the time of receiving the reported intervention, yet without the use of stratified analysis, were excluded. 

### 2.3. Data Extraction and Summary

Using the above search strategy and inclusion and exclusion criteria, two authors independently screened the titles and abstracts of the extracted studies, and selected the articles for inclusion in the review. The resultant list of included articles was confirmed by a third author. The full-text of the included articles was further examined by the three authors to confirm the eligibility of the articles to be included in the review. 

Upon finalization of the list of included articles, two authors independently performed data extraction, which was subjected to verification by one author to ensure accuracy. These data included settings of the reported interventions, characteristics of the participants, content of intervention, assessed outcomes of interest, data collection time points, instruments used for outcome assessments, and study findings. Disagreements between the authors on the extracted data were resolved through discussion until a consensus was made.

In this review, the significance of intervention effects on the outcomes of interest was indicated with *p* values. 

### 2.4. Critical Appraisal 

The assessment of the methodological quality of the selected studies was performed using “The Quality Assessment Tool for Quantitative Studies” in the Effective Public Health Practice Project (EPHPP). The methodology on how the assessment tool is used for critical appraisal of the included studies was previously described [23]. Briefly, a rating of either strong, moderate or weak was given for each study on six categories involved in the assessment, namely selection bias, study design, confounders, blinding, data collection methods and withdrawals and dropouts. The elements assessed in each of these categories were described previously [23]. Such ratings were performed based on the assessment criteria set out by the EPHPP [24]. Based on the rating for each category, a global rating was given. A strong global rating indicates the study received no weak ratings in any category and has four or more strong-rated categories. A study was assigned a global rating of “moderate” if it possessed one weak category and/or fewer than four strong-rated categories. A study with more than one category having a weak rating was given a “weak” global rating. This critical appraisal was performed by three authors independently. Disagreements in rating assignments were resolved through discussion between the three authors.

## 3. Results

### 3.1. Search Results

**Figure 1:**
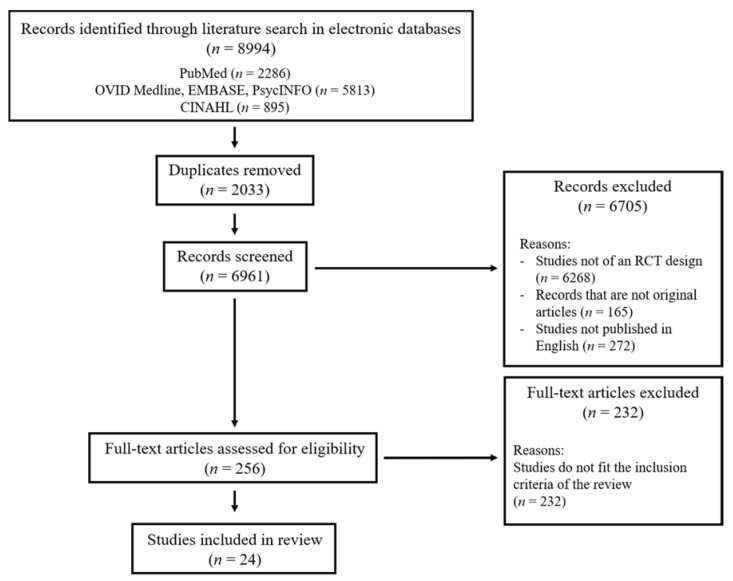
The PRISMA Diagram.

The PRISMA diagram is presented as Figure 1. Briefly, the search strategy (Table 1) enabled the identification of 8994 articles. After the removal of 2033 articles retrieved in duplications in multiple databases, we screened the abstracts of the remaining 6961 articles. We excluded 6268 articles not reporting an RCT, 165 articles that were not original articles and 272 articles not published in English. A total of 232 articles were further excluded as they did not report an intervention or they did not involve the participant type and/or outcome measures indicated in the inclusion criteria. The search resulted in the inclusion of 24 articles in the present review. 

### 3.2. Methodological Quality of Included Studies

Fifty-four percent (*n* = 13) of included studies were given an overall rating as weak, 25% (*n* = 6) as moderate, and 21% (*n* = 5) as strong. Around 67% of the included studies (*n* = 16) were rated moderate for selection bias. These studies utilized an appropriate sampling method to generate a representative sample of the population. A number of studies did not report the number of participants approached during subject recruitment, making it impossible to determine the percentage of participants who provided their consent to study participation. 

Considerable proportion of the included studies were rated as strong in the study design (*n* = 14, 58%) where most studies utilized an appropriate randomization technique. Eighty-eight percent of studies (*n* = 21) were rated as strong in the confounders category, where they either demonstrated no significant between-group differences in baseline characteristics or confounders were addressed in the case where such differences were observed. Additionally, eight studies (33%) were rated as strong in the blinding category, where they involved participants and assessors who were blinded to the treatment allocation. Most of the studies reported a strong data collection method (*n* = 22, 92%), with the data collection tools used in these studies demonstrated to be reliable and valid. Fifty-eight percent (*n* = 14) of studies were rated as strong in the withdrawals and dropouts category, where they reported that at least 80% of the participants had completed the study. Table 2 shows a tabular summary of the methodological quality ratings of each included study. In summary, 46% of the included studies were either rated strong or moderate in the appraisal. Weaknesses of the methodological quality among these studies were primarily contributed by: (1) the impossibility of blinding the participants and/or outcome assessors owing to the nature of the intervention; and (2) the lack of reporting on the number of participants approached during subject recruitment and the method of randomization conducted.

### 3.3. Effects of Interventions on Patient Outcomes

The characteristics of the included studies, including the content of the interventions reported, are summarized in reverse chronological order in Table 3. Our review identified pharmacological interventions, four major types of non-pharmacological interventions (physical activity interventions, psychotherapeutic interventions, dietary interventions and acupuncture interventions) and other miscellaneous types of interventions that have previously been developed to aid management of adjuvant endocrine therapy-induced symptoms among breast cancer survivors. This section of the review summarizes the findings of the studies reporting their effect on the alleviating the various groups of adjuvant endocrine therapy-induced symptoms as menopausal symptoms, impaired sexual function, joint symptoms, fatigue and sleep disturbance, as well as the enhancement of survivors’ QOL, HRQOL and functional status.

#### 3.3.1. Menopausal Symptoms

Menopausal symptoms investigated in this review include hot flashes, night sweats and vaginal atrophy. An overview of the intervention effect on menopausal symptoms is provided in Table 4.

##### Hot Flashes

Five studies reported the effect of pharmacological interventions on addressing hot flashes among the participants [25,26,27,28,29]. While earlier studies showed that pharmacological interventions using clonidine could lead to significant reduction in hot flashes score, frequency and intensity [28,29], other studies reported non-significant differences (*p* ≥ 0.54) in the changes of these parameters between the intervention and control group at pre- and post-intervention [25,26,27]. Notably, both the intervention and control participants reported a decrease in these parameters from baseline to post-intervention [25,26], which demonstrates why non-significant differences were observed. Interestingly, in a cross-over study on a pharmacological intervention using the anti-depressant sertraline, significant between-group differences were observed in hot flashes frequency and hot flashes score after cross-over at Week 12 of the intervention, despite the lack of such difference before the cross-over at Week 6 [27]. This suggests that the intervention may take longer for the exhibition of its alleviation effect on hot flashes. These data demonstrate that certain pharmacological products, which have not been intended for use to treat menopausal symptoms, could still be of value for use in pharmacological interventions to alleviate hot flashes among breast cancer patients undergoing adjuvant endocrine therapy.

Moreover, Mann et al. [30] reported the effect of a cognitive behavioral therapy on the perceived burden of hot flashes. The intervention exhibited a significant effect on reducing such burden at Week 9 and Week 26 of the study.

##### Night Sweats

Mann et al. [30] also examined the effect of the cognitive behavioral therapy on reducing the perceived burden on night sweats. Similar to hot flashes burden, the intervention exhibited an alleviation effect on perceived night sweats burden at both Week 9 and Week 26 of the study.

##### Vaginal Atrophy

Keshavarzi et al. [31] reported the use of a vitamin D/vitamin E vaginal suppository intervention in addressing vaginal atrophy among breast cancer survivors. Compared to controls, patients receiving the intervention had significantly greater decrease in perceived levels of vaginal atrophy, as assessed by the genitourinary atrophy score, throughout the eight-week intervention (*p* ≤ 0.017). No longer-term assessment on patient outcomes was conducted to assess whether there are any long-term effects of the intervention.

#### 3.3.2. Sexual Dysfunction

Table 4 presents a summary of the effects of interventions on sexual problems among breast cancer survivors. 

De Sousa Vieira et al. [25] reported that a pharmacological intervention using a medicinal plant extract (Guarana) did not have significant effect on increasing sex drive among the participants. Likewise, an exercise training intervention did not exhibit significant enhancement of sexual functioning or sexual enjoyment among participants [32]. A multimodal intervention, comprising sexual counselling and the use of vaginal moisturizers, lubricants and/or dilator, contributed to a greater improvement of sexual function and reduction of sexual distress at post-intervention among intervention participants (*p* ≤ 0.04) [33]. Moreover, intervention participants tended to have a reduced level of dyspareunia compared with control participants at post-intervention (*p* = 0.07). These data indicate that a multimodal intervention combining the delivery of sexual counselling and vaginal pain relief could be considered for use in addressing sexual problems among breast cancer survivors.

#### 3.3.3. Joint Symptoms

Ten studies reported the effect of their respective interventions on addressing joint symptoms, including joint pain and joint stiffness, among breast cancer survivors undergoing adjuvant endocrine therapy (Table 5).

Henry et al. [34] was the only group to investigate the effect of pharmacological interventions on joint symptoms. The study demonstrated that an intervention involving the intake of duloxetine, a known anti-depressant, could alleviate joint pain and joint stiffness and reduce pain interference score, where significant between-group differences were observed in these outcomes throughout the intervention. However, the intervention lacks a long-term effect in the alleviation of joint pain and stiffness, with no significant between-group differences in the aforementioned outcomes observed at 12 weeks after intervention discontinuation.

Four studies reported the effect of physical activity interventions on addressing joint symptoms. Three of the studies, which report a home-based walking program and physical activity behavior change intervention that lasted 6–12 weeks, demonstrated no significant effect on alleviating joint pain [35,36,37], although one reported a trend for a decrease in joint pain among the intervention participants [35]. Two reported no significant between-group differences in physical dysfunction of joints (*p* ≥ 0.09) [36,37]. In contrast, an exercise intervention lasting for one year was effective in alleviating joint pain, with significant between-group differences observed throughout the intervention for worst joint pain scores and joint pain severity [38]. Inconsistency is observed between all studies regarding the effect of exercise interventions on perceived joint stiffness, where only one study reported differential between-group outcomes on the Western Ontario and McMaster Universities Osteoarthritis Index (WOMAC) score [38]. Overall, physical activity interventions do not appear to be effective in alleviating joint pain or dysfunction among breast cancer patients undergoing adjuvant endocrine therapy, but they are potentially useful in addressing joint stiffness. Moreover, a physical activity intervention that is longer in duration could potentially be useful in addressing both joint pain and stiffness.

One study investigated the effect of a dietary intervention involving omega-3 fatty acid intake on joint symptoms [39]. Hershman et al. demonstrated a lack of effect of this intervention on reducing overall pain, joint pain and joint stiffness of participants. The intervention did not have any effect on the level of interference on daily activities caused by the above symptoms either (*p* ≥ 0.12). 

Three studies reported the effect of acupuncture on addressing joint pain and stiffness [40,41,42]. Conflicting data were reported by these studies. While Hershman et al. reported significant between-group differences of worst joint pain scores, joint pain severity and worst joint stiffness scores at post-intervention [40], Oh et al. observed otherwise [41]. Crew et al. [42] reported that, while significant between-group difference was observed when the above parameters were assessed using Brief Pain Inventory-Short Form (BPI-SF), such significance difference was no longer observed when Western Ontario and McMaster Universities Osteoarthritis Index (WOMAC) was used for outcome measurement. Overall, firm conclusions cannot be drawn on whether acupuncture interventions can effectively address joint pain and joint stiffness.

Baker et al. [43] examined the effectiveness of a whole-body vibration intervention on joint symptom management among breast cancer survivors. This intervention was found to have no significant effect on alleviation of fatigue, joint pain, joint stiffness and physical dysfunction of joints (*p* ≥ 0.06). Additionally, the intervention had no effect on the improvement of participants’ functional ability.

Overall, while a number of studies investigated the effect of interventions on addressing joint symptoms among breast cancer survivors undergoing adjuvant endocrine therapy, the inconsistency of these findings based on intervention types results in difficulties in drawing firm conclusions on which intervention type is the most appropriate for this purpose.

#### 3.3.4. Fatigue

Intervention effects on fatigue among breast cancer survivors undergoing adjuvant endocrine therapy were investigated in seven studies (Table 6). 

Zhao et al. [44] reported the only study that examined the effect of pharmacological interventions on fatigue levels. They found that the intake of spore powder from *G. lucidum* would lead to a significant improvement of fatigue (*p* < 0.01). This finding was further supported by the observation that such an intervention could significantly lower participants’ serum TNF-alpha and IL-6 levels (*p* < 0.01), which are shown to have linear correlations with cancer-related fatigue.

Two studies assessed the effects of physical activity interventions on fatigue levels, and conflicting data were obtained. While a significant difference in fatigue levels (*p* = 0.001) was demonstrated between groups and over time by Paulo et al. [32] who reported an exercise program, Nyrop et al. [35] who reported a home-based walking program reported no significant between-group differences at post-intervention. It is therefore difficult to draw firm conclusions on the effect of physical activity interventions on fatigue levels.

Using a combination of instruments for fatigue assessment, Peppone et al. showed that yoga intervention involving mindfulness exercises could reduce fatigue symptoms significantly (*p* = 0.001) among the participants having received the intervention [45], demonstrating the potential of similar psychotherapeutic interventions to address this symptom.

Mao et al. conducted the sole study in this review to report the effect of acupuncture intervention on fatigue [46]. They reported that participants who received electro-acupuncture exhibited a significantly greater extent of improvement in fatigue levels at post-intervention (*p* = 0.0095), and this effect was sustained at four weeks post-intervention (*p* = 0.022). 

The effect of a whole-body vibration intervention and neuromuscular taping intervention on reducing fatigue among the survivors was assessed by Baker et al. [43] and Conejo et al. [47], respectively. The whole-body vibration intervention exhibited no significant effect on improving fatigue at post-intervention (*p* = 0.079). The neuromuscular taping intervention appeared to have significantly alleviated fatigue, albeit a need for a longer duration post-intervention to take effect. Significant between-group differences in fatigue severity was only observed at five weeks after receiving this intervention.

#### 3.3.5. Sleep Disturbance

Seven studies involved sleep disturbance as an outcome for assessing the effectiveness of interventions for breast cancer survivors undergoing adjuvant endocrine therapy (Table 7). 

Four studies examined the effect of physical activity interventions on addressing sleep disturbance and/or improving sleep quality [32,36,37,48]. While two of the studies reported a significantly greater decrease in sleep disturbance (*p* ≤ 0.04) and improvement in sleep quality (*p* = 0.002) immediately post-intervention [32,48], the two other studies reported a lack of significant difference on these parameters [36,37]. Notably, the physical activity behavior change intervention reported by Rogers et al. [48] no longer exhibited a beneficial effect on alleviating sleep disturbance and improving sleep quality at three-month post-intervention, suggesting that the intervention may not exhibit long-term effectiveness, and it prompts a need for continuous practice of the intervention for it to take effect.

Cognitive behavioral therapy, a type of psychotherapeutic interventions, was shown by Mann et al. [30] to significantly improve participants’ sleep quality at post-intervention (*p* < 0.001), and this beneficial effect persisted for a further 17 weeks (*p* < 0.05). These data suggest the potential of this therapy to be implemented for addressing sleep problems among breast cancer survivors.

In contrast, acupuncture intervention may not be as effective in addressing sleep problems. In a study involving an electro-acupuncture intervention reported by Mao et al. [46], the authors reported a lack of significant difference in the improvement of the Pittsburgh Sleep Quality Index (PSQI) score between groups (*p* ≥ 0.058) at four- and eight-week follow-up. These data show that such intervention is unlikely to be appropriate for improving sleep quality among breast cancer survivors in both the short and long term.

Likewise, the neuromuscular taping intervention appeared ineffective in addressing sleep problems, as evidenced by the lack of significant between-group difference in the perceived levels of insomnia among the participants at both immediate post-intervention and four weeks post-intervention [47].

#### 3.3.6. QOL/HRQOL 

Thirteen studies reported the effects of their respective intervention on QOL or HRQOL outcomes of breast cancer survivors undergoing adjuvant endocrine therapy, and two studies reported that on functional ability of these survivors (Table 8). 

There were five studies reporting different pharmacological interventions on survivors’ QOL. Three of the studies demonstrated that such interventions could lead to an improvement on this outcome. Zhao et al. [44] reported a significantly greater extent of improvement in multiple domains of QOL among participants who took spore powder of *G. lucidum* compared to control. Functional QOL was also reported by Henry et al. to have been more significantly improved among participants who took duloxetine [34]. An intervention involving clonidine intake could also lead to a short-term improvement on patients’ QOL (*p* ≤ 0.022) at four- and eight-week follow-up, but this effect was no longer observed at Week 12 (*p* > 0.20) [28]. In contrast, pharmacological interventions involving the use of sertraline or Guarana appeared to have no effect on patients’ QOL [25,27].

Four studies reported the effect of physical activity interventions on QOL outcomes. Participants who received the supervised combined exercise training intervention were reported to exhibit a greater extent of improvement on this outcome [32], suggesting the effectiveness of an intervention combining resistance, aerobic and stretching exercises in QOL improvement. Rogers et al. [36,37] also showed in a pilot study that the physical activity behavior change intervention comprising supervised exercise, home-based exercise and physical activity counselling sessions exhibited significant effects in improving social well-being and overall QOL, although such effect was only exhibited at three months post-intervention. In contrast, a home-based walking program appeared to have no effect in QOL improvement, as shown by the insignificant differences of FACT-G scores at post-intervention between participants who received the program and the control counterparts [35].

Cognitive behavioral therapy was also shown to have some beneficial effect on survivors’ HRQOL [30]. Participants who received this therapy were reported to have a significantly greater extent of improvement on general health, physical functioning and social functioning than controls, although significant improvement for the latter two outcomes was only observed later at Week 26 of the study. 

Conflicting findings were obtained regarding the effect of acupuncture interventions on survivors’ QOL. While implementation of an acupuncture intervention would enable participants to exhibit a significantly greater extent of QOL improvement [42], that of an electro-acupuncture intervention failed to do so [41]. Note that, while the significant effect of QOL improvement exhibited by the acupuncture intervention did not persist in the long term [42], there was a trend for increased improvement of physical functioning among participants who received the electro-acupuncture intervention at a longer-term follow-up [41]. 

Finally, the neuromuscular taping intervention was found to be effective in the improvement in participants’ perceived global health status and QOL [47]. A more significant improvement in these parameters was only observed among the intervention participants at five weeks after receiving the intervention (*p* = 0.005) and not immediately post-intervention. This suggests a need for a longer duration for the intervention to take effect.

#### 3.3.7. Functional Ability

Two studies, reporting a home-based walking program and a whole body vibration intervention, examined the effect of their respective intervention on functional ability of breast cancer survivors undergoing adjuvant endocrine therapy (Table 8). While intervention participants who received the home-based walking program experienced a significantly greater reduction in their difficulty with activities involved in their daily living [35], those who received the whole body vibration intervention reported no significant improvement in their functional ability, as measured by their ability to rise from a chair and climbing stairs [43].

## 4. Discussion

Overall, pharmacological interventions were reported to result in a perceived improvement in patients’ QOL, suggesting a potential for using pharmacological products in enhancing patients’ well-being. We also noted that these interventions appear to have different effects on symptoms such as hot flashes. This is not surprising because these reported interventions utilized different pharmacological products. For example, clonidine and potentially sertraline were shown to minimize hot flashes in earlier studies [27,28,29]. However, homeopathic medicine and Guarana extracts were reported to have no effect on alleviation of this symptom [25,26]. Indeed, one might also argue that the methodological quality of these studies could contribute to these conflicting findings. However, studies showing a positive effect of their interventions on hot flashes appeared to have mixed ratings. Among the three studies [27,28,29] that reported such positive effect, two were rated weak and one has a strong global rating. Meanwhile, another study [26] did not report any effect of the intervention on hot flashes, despite its strong global rating of its methodological quality. Thus, differences in quality ratings of the studies are unlikely to be a factor that can account for the discrepancies of findings. 

Interestingly, clonidine, a pharmacological product for treatment of hypertension, can also exhibit alleviating effect on hot flashes. This finding prompts that pharmacological products intended for use to treat a certain non-adjuvant endocrine therapy-induced symptom could also have beneficial effects on addressing common symptoms induced by this therapy. Such findings suggest a value for conducting further studies to explore the beneficial effect of widely-used cancer palliative medications known to have minimal side effects in addressing various symptoms [49]. This would help establish the most suitable medication plan in pharmacological interventions for supportive care among patients with optimal effectiveness. 

In addition, intake of traditional Chinese medicine such as the spore powder of *G. lucidum* would have beneficial effects in the management of cancer-associated symptoms including fatigue and improving QOL [44]. It is likely that the bioactive compounds present in *G. lucidum*, such as triterpenoids [50], could play a role in mediating these effects. Indeed, triterpenoids were shown to exhibit anti-oxidative effects [51], which are known to be beneficial in fatigue reduction [52]. Moreover, triterpenoids would also play a role in fatigue reduction through modulation of cytokine expression [52]. The findings by Zhao et al. [44] may therefore make a case for the exploration of dietary or medicinal products containing anti-oxidative and immunomodulatory bioactive compounds that can be used in interventions for addressing cancer-associated symptoms. 

Despite the potential of pharmacological interventions in symptom management among breast cancer survivors, these interventions were also reported to induce undesirable side effects. For example, although clonidine was shown to be useful for addressing hot flashes among patients, it was also reported to contribute to symptoms including drowsiness, sleep difficulties, dry mouth and constipation [28,29]. Likewise, despite its effectiveness in treating fatigue, pharmacological intervention involving the intake of *G. lucidum* could also cause mild symptoms such as dry mouth and dizziness [44]. In general, side effects of drugs used should be taken into account when pharmacological interventions are designed to manage particular symptoms.

Our review also demonstrates that certain non-pharmacological interventions, which were suggested to be less prone to the occurrence of undesirable side effects [53], can be effective in symptom management and enhancing QOL. For example, psychotherapeutic interventions such as yoga could effectively reduce fatigue among breast cancer survivors, while cognitive behavioral therapy could help address sleep disturbance, enhance survivors’ QOL, and reduce their perceived burden of hot flashes and night sweats. Acupuncture and electro-acupuncture interventions can likewise address fatigue and potentially joint pain and stiffness. Although conflicting findings were noted between studies reporting the effect of acupuncture interventions on joint symptoms [40,41], we believe that such discrepancies could be contributed by the different sample size used in these studies. While the study with 226 participants indicated a significant effect of an acupuncture intervention on reducing joint symptoms [40], the study with only 29 participants reported otherwise [41]. Owing to the effect of sample sizes on the significance of between-group differences of clinical effects, future studies should consider the use of larger sample sizes for the examination of the effects of interventions on patient outcomes. 

Conflicting data were obtained on the effect of physical activity interventions on all of the assessed outcomes. In our review, the included studies reported physical activity interventions involving different types of exercises including aerobic exercises, walking, use of cycle ergometers and home-based exercises. One may perceive that the intensity of exercise involved in the intervention could be a factor for the discrepancies observed, as evidenced by the difference in the effect of a home-based walking program and a program combining resistance and aerobic exercises on joint pain [35,38]. However, as reported in a recent meta-analysis, interventions involving different types of exercises were consistently shown to reduce pain, fatigue and insomnia among patients of various cancer types [54]. This finding prompts that intensity of exercise involved in an intervention is unlikely to have affected intervention effectiveness. One may also argue that participants’ adherence to the physical activity interventions could affect patient outcomes. Indeed, Paulo et al. [32] and Irwin et al. [38] both reported good participant adherence to the intervention, and that their intervention could lead to significant improvement of outcomes including sleep, fatigue, joint pain and QOL. However, as demonstrated by Rogers et al. [37], even though participants’ adherence was reported to be over 95%, patient outcomes such as joint pain were not significantly improved. It is therefore unlikely that participants’ adherence to the intervention could play a significant role in modifying intervention effectiveness. We therefore hypothesize that the aforementioned discrepancies of findings are due to the duration of the reported interventions. For example, while shorter physical activity interventions were found not to be effective in addressing joint pain, a year-long physical activity intervention reported by Irwin et al. [38] appeared to be effective in doing so. Likewise, while longer interventions lasting nine months were found to have significant effect on the reduction of patients’ fatigue [32], the six-week program reported by Nyrop et al. appeared to have no effect [35]. These data suggest that implementation of interventions that last longer could be more effective in symptom management, and future studies should take intervention duration into account to enhance intervention effectiveness.

It is also worth noting that non-pharmacological interventions that target specific symptoms were shown to be particularly effective in symptom management and improving patients’ well-being. For example, an intervention that specifically targets vaginal symptoms, such as vitamin D/E vaginal suppository intervention, can effectively alleviate vaginal atrophy, a common menopausal symptom among breast cancer patients [31]. Likewise, neuromuscular taping, an intervention that specifically targets body pain, was shown to be effective in pain relief among patients, in addition to the alleviation of fatigue and improvement of QOL [47]. These observations suggest that the development of multimodal interventions comprising multiple intervention strategies that were known to target a particular symptom could be more effective for symptom management.

This review has three limitations. First, only studies published in English were included in this review. Therefore, studies that could contribute further useful data to this review, yet not published in English were neglected, thereby limiting the comprehensiveness of this review. Second, the majority of the included studies have a weak overall rating in the appraisal on their methodological quality. With each of the categories in the appraisal having a potential in causing bias, the review findings have to be interpreted with caution. Third, several included studies utilized a small sample of about 30 participants. These studies would therefore be prone to small sample bias, a source for false negative results that lead to difficulties in drawing firm conclusions regarding the effects of the reported interventions [55].

### Implications

Our review has suggested that various types of interventions, pharmacological and non-pharmacological alike, could be of value for implementation to help breast cancer patients undergoing adjuvant endocrine therapy to manage symptoms and improve QOL. Moreover, they suggest the benefit of using a multimodal approach in intervention design for addressing multiple adjuvant endocrine therapy-induced symptoms. Our findings thus provide an informative basis for further research into the effective approaches that would ameliorate symptoms induced by adjuvant endocrine therapy. This may improve adherence in breast cancer survivors undergoing adjuvant endocrine therapy, enabling it to be more effective in preventing cancer recurrence.

## 5. Conclusions

Our review shows that both pharmacological and non-pharmacological interventions can be effective for symptom management among breast cancer survivors undergoing adjuvant endocrine therapy, potentially enhancing their QOL. Nevertheless, firm conclusions cannot be drawn from the review over the types of interventions that are most optimal for supportive care of breast cancer survivors and highlights the need for a multimodal approach. This is due to the discrepancies in the findings between the included studies over the significance of the between-group differences in the extent of improvement of symptoms and QOL. Despite this, our review suggests that a longer duration in physical activity interventions could be more effective for symptom management. Further studies on whether the duration of physical activity interventions, and potentially that of other reported non-pharmacological interventions such as acupuncture, would be associated with intervention effectiveness on improving symptoms and QOL are therefore recommended. Moreover, these studies should involve the use of larger sample sizes for the generation of more reliable results. Overall, these studies would help contribute to the evidence of the utility of these interventions for symptom management among survivors undergoing these therapies.

## Figures and Tables

**Table 1:** The Search Strategy.

“Breast cancer” OR “carcinoma” OR “neoplasm” OR “malignancy”
AND
“survivorship” OR “survivor” OR “survivors” OR “patient” OR “patients”
AND
“Tamoxifen” OR “Aromatase inhibitor” OR “endocrine therapy” OR “hormone therapy” OR “hormonal therapy”
AND
“Intervention” OR “therapy” OR “program” OR “programme” OR “pharmacological” OR “non-pharmacological”
AND
“quality of life” OR “well-being” OR “well being” OR “symptom” OR “symptoms” OR “side effects” OR “psychological” OR “psychosocial” OR “stress” OR “distress” OR “anxiety” OR “depression” OR “sexuality” OR “sexual function” OR “sexual dysfunction” OR “hot flash” OR “hot flush” OR “vaginal dryness” OR “pain” OR “fatigue” OR “sleep disturbance” OR “joint pain” OR “joint stiffness” OR “functional ability”

**Table 2:** The methodological quality of the included studies.

Author/Year [25,26,27,28,29,30,31,32,33,34,35,36,37,38,39,40,41,42,43,44,45,46,47,48]	Methodological Quality Rating (EPHPP)						
	Selection Bias	Study Design	Confounders	Blinding	Data Collection Method	Withdrawals and Dropouts	Overall
Advani et al., 2017 [33]	Moderate	Strong	Strong	Weak	Strong	Strong	Moderate
Baker et al., 2018 [43]	Moderate	Strong	Strong	Moderate	Strong	Strong	Strong
Conejo et al., 2018 [47]	Moderate	Strong	Strong	Weak	Strong	Strong	Moderate
Crew et al., 2007 [42]	Moderate	Weak	Weak	Weak	Strong	Strong	Weak
de Sousa et al., 2019 [25]	Moderate	Weak	Strong	Strong	Strong	Moderate	Moderate
Goldberg et al., 1994 [29]	Weak	Weak	Strong	Strong	Weak	Weak	Weak
Henry et al., 2018 [34]	Weak	Weak	Strong	Strong	Strong	Strong	Weak
Hershman et al., 2015 [39]	Weak	Weak	Strong	Strong	Strong	Moderate	Weak
Hershman et al., 2018 [40]	Moderate	Weak	Weak	Weak	Strong	Strong	Weak
Heudel et al., 2019 [26]	Moderate	Strong	Strong	Strong	Strong	Strong	Strong
Irwin et al., 2015 [38]	Moderate	Strong	Strong	Weak	Strong	Strong	Weak
Keshavarzi et al., 2019 [31]	Moderate	Strong	Strong	Strong	Strong	Strong	Strong
Kimmick et al., 2006 [27]	Weak	Weak	Strong	Strong	Strong	Moderate	Weak
Mann et al., 2012 [30]	Moderate	Strong	Strong	Moderate	Strong	Strong	Strong
Mao et al., 2014 [46]	Weak	Strong	Strong	Weak	Strong	Strong	Weak
Nyrop et al., 2017 [35]	Moderate	Weak	Weak	Weak	Strong	Moderate	Weak
Oh et al., 2013 [41]	Moderate	Strong	Strong	Weak	Strong	Strong	Moderate
Pandya et al., 2000 [28]	Moderate	Strong	Strong	Strong	Strong	Moderate	Strong
Paulo et al., 2019 [32]	Moderate	Weak	Strong	Weak	Strong	Moderate	Weak
Peppone et al., 2015 [45]	Weak	Strong	Strong	Weak	Weak	Weak	Weak
Rogers et al., 2009 [36]	Moderate	Strong	Strong	Weak	Strong	Strong	Moderate
Rogers et al., 2009 [37]	Moderate	Strong	Strong	Weak	Strong	Strong	Moderate
Rogers et al., 2017 [48]	Weak	Strong	Strong	Moderate	Strong	Weak	Weak
Zhao et al., 2012 [44]	Weak	Weak	Strong	Weak	Strong	Weak	Weak

**Table 3:** Characteristics of the included studies.

Author/Year/Country	Settings	Participants/Sample Size	Intervention Type	Intervention	Assessed Outcomes of Interest/Data Collection Time Points	Instruments for Outcome Assessments
de Sousa Vieira et al., 2019 [25]; Brazil	Not specified	Breast cancer patients (stage unknown) undergoing adjuvant hormone therapy using either tamoxifen or anastrozole. *N* = 40 Intervention: 20 Control: 20 Duration of adjuvant endocrine therapy undergone by subjects: All participants: At least 3 months (not specified for each group)	Pharmacological intervention	Pharmacological intervention with tablets of a medicinal plant extract (Paullinia cupana or Guarana) Intervention: Intake of tablets containing 37.5 mg of an active ingredient of guarana (PC-18) twice daily for 4 weeks. Control: Intake of placebo tablets for 4 weeks	The frequency and severity of hot flashes Sexual dysfunction Depression, QOL Data collected at: Baseline Post-intervention	Daily hot flashes diary Arizona Sex Experience Scale (ASEX) The Beck Depression Scale European Organization for Research and Treatment of Cancer Core Quality of Life Questionnaire C30
Heudel et al., 2019 [26]; France	Not specified	Patients with localized breast cancer (stage unknown), receiving adjuvant endocrine therapy with tamoxifen or aromatase inhibitors *N* = 138 Intervention: 65 Control: 73 Duration of adjuvant endocrine therapy undergone by subjects: All participants: At least 1 month (not specified for each group)	Pharmacological intervention	Pharmacological intervention with a homeopathic medicine named Actheane® Intervention: Intake of Actheane® tablets twice a day for a period of 8–10 weeks. Control: Intake of placebo tablets, twice a day for a period of 8–10 weeks.	Hot flashes score Frequency of hot flashes Hot flashes impact on QOL Data collected at: Baseline 4 weeks after randomization (T1) 8 weeks after randomization (T2)	Hot flush self-report diary Hot Flash Related Daily Interference Scale
Keshavarzi et al., 2019 [31]; Iran	Local breast clinic	Patients with stage I–II breast cancer, undergoing tamoxifen therapy *N* = 96 Vitamin D group: 32 Vitamin E group: 32 Placebo group: 32 Duration of adjuvant endocrine therapy undergone by subjects: Vitamin D group: 23.6 ± 17.5 months Vitamin E group: 30.4 ± 21 months Control group: 16.6 ± 12.5 months	Miscellaneous intervention	Vitamin D/vitamin E vaginal suppository intervention Intervention: Participants were provided with vaginal suppositories supplemented with either 0.025 mg vitamin D or 1 mg vitamin E, and were instructed to insert one suppository into the vagina every day before bedtime, over the course of 8 weeks. Telephone reminders were given every 3 days. Control: The use of vaginal suppositories supplemented with placebo, instead of vitamin D/E vaginal suppositories.	Level of vaginal atrophy Data collected at: Baseline 2 weeks after start of intervention 4 weeks after start of intervention 8 weeks after start of intervention	The genitourinary atrophy self-assessment tool
Paulo et al., 2019 [32]; Brazil	Not specified	Breast cancer survivors previously diagnosed with stage I–IIIA cancer, undergoing aromatase inhibitor therapy *N* = 36 Intervention: 18 Control: 18 Duration of adjuvant endocrine therapy undergone by subjects: Intervention group: 19.3 ± 8.3 months Control group: 17.9 ± 11.2 months	Physical activity intervention	Supervised combined exercise training intervention Intervention: Participation in a 9-month exercise program, with sessions held 3 times a week, containing resistance training exercises, aerobic exercises and stretching exercises Attendance to 90-min health education lectures once a month, with topics on breast cancer, health promotion, quality of life, physical activity, well being and mental health Control: Participation of 45-min sessions of stretching and relaxation exercises twice a week, over the course of 9 months.	QOL Pain Fatigue Sleep disturbance Sexual functioning Data collected at: Baseline 12 weeks after start of intervention (T1) 24 weeks after start of intervention (T2) 36 weeks after start of intervention (T3)	European Organization for Research and Treatment of Cancer Core Quality of Life Questionnaire C30 (EORTC-CLC-C30) European Organization for Research and Treatment of breast cancer module Short Form 36 Health Survey (SF-36)
Baker et al., 2018 [43]; Australia	Local institution or hospital department	Breast cancer patients (stage unknown) undergoing aromatase inhibitor therapy *N* = 31 Intervention: 14 Control: 17 Duration of adjuvant endocrine therapy undergone by subjects: Intervention group: 19 months (median) Control group: 3 months (median)	Miscellaneous intervention	Whole body vibration intervention Intervention: Attendance to sessions involving exposure to low-frequency and low-magnitude whole body vibration via a vibration platform. Each session lasted for 20 min, and was held 3 times a week for a duration of 12 weeks. Control: Usual care	Functional ability Fatigue Joint symptoms Data collected at: Baseline Immediate post-intervention	Chair rise and stair climb (objective measure of functional ability) Functional Assessment of Cancer Therapy–Fatigue Western Ontario and McMaster Universities Arthritis Index
Conejo et al., 2018 [47]; Spain	Not specified	Breast cancer survivors previously diagnosed with stage I–IIIA cancer, undergoing aromatase inhibitor therapy *N* = 40 Intervention: 20 Control: 20 Duration of adjuvant endocrine therapy undergone by subjects: Intervention group: 15.25 months (mean) Control group: 19.55 months (mean)	Miscellaneous intervention	Neuromuscular taping intervention Intervention: Application of strips of neuromuscular taping over the body parts where pain was felt for 7 days Provision of health advice, focusing on active lifestyles Control: Sham neuromuscular taping over body parts where pain was felt for 7 days Provision of health advice, focusing on active lifestyles	Mood state QOL Fatigue Pain Sleep disturbance Data collected at: Baseline 1 week after start of intervention (T1) 5 weeks after start of intervention (T2)	Profile of Mood States European Organization for Research and Treatment of Cancer Core Quality of Life Questionnaire C30 (EORTC-CLC-C30) QuickPIPER C-reactive protein and creatine kinase levels (objective measurement of fatigue and insomnia) Visual analogue scale
Henry et al., 2018 [34]; USA	Not specified	Breast cancer patients (stages I–III), receiving aromatase inhibitor therapy *N* = 299 Intervention: 150 Control: 149 Duration of adjuvant endocrine therapy undergone by subjects: All participants: 47.9 ± 36.3 weeks (not specified for each group)	Pharmacological intervention	Pharmacological intervention with Duloxetine Intervention: Intake of 1 capsule containing 30 mg duloxetine per day for 1 week. Intake of 2 of the capsule described above for the subsequent 11 weeks. Thereafter, patients took 1 capsule daily for one further week. Control: Intake of placebo containing sugar spheres over the schedule described above	Joint pain and stiffness QOL Depression Data collected at: Baseline Week 2 post-randomization (T1) Week 6 post-randomization (T2) Week 12 post-randomization (T3) Week 24 post-randomization (T4)	Brief Pain Inventory (BPI) Western Ontario and McMaster Universities Osteoarthritis scale The Global Rating of Change Scale (GRCS) The Functional Assessment of Cancer Therapy-Endocrine Scale Trial Outcome Index The brief Patient Health Questionnaire-9
Hershman et al., 2018 [40]; USA	11 Academic and community sites—sites not specified	Breast cancer patients, undergoing aromatase inhibitor therapy *N* = 226 Intervention: True acupuncture = 110 Control Groups: Sham Acupuncture = 59 Waiting list = 57 Duration of adjuvant endocrine therapy undergone by subjects: All participants: At least 1 month (not specified for each group)	Acupuncture intervention	Acupuncture intervention Intervention Group: 12 sessions of true acupuncture over the first 6 weeks. Each session lasted for 30-45 min. Six more weekly sessions of true acupuncture were held over the next 6 weeks. Control Group 1: Sham Acupuncture Sessions of sham acupuncture based on the schedule presented above. Control Group 2: The waitlist control group Delayed treatment using true acupuncture received by intervention group	Joint pain and joint stiffness Sexual function Distress about sexual problems Data collected at Baseline 6 weeks after randomization (T1) 12 weeks after randomization (T2) 16 weeks after randomization (T3) 20 weeks after randomization (T4) 24 weeks after randomization (T5) 52 weeks after randomization (T6)	The Brief Pain Inventory Short Form Western Ontario and McMaster Universities Osteoarthritis Index Functional assessment of Cancer Therapy-Endocrine Symptoms The Patient-Reported Outcomes Measurement Information System (PROMIS) Pain Impact-Short Form
Advani et al., 2017 [33]; USA	Not specified	Localized breast cancer patients (early stage), receiving adjuvant endocrine therapy with aromatase inhibitor *N* = 57 Control group: Usual Care Group: 21 Intervention groups: Active Group-H:18 Active Group-P:18 Duration of adjuvant endocrine therapy undergone by subjects: All participants: Less than 4 weeks (not specified for each group)	Multimodal intervention	A multimodal intervention involving sexual counseling, and use of vaginal moisturizers, lubricants, and/or dilator A booklet, Why It Is Important to Take Your Aromatase Inhibitor, was given to both intervention and control participants in the study, encouraging adherence to endocrine therapy and provision of information on self-help strategies for symptom management. Intervention: Provision of one brand of vaginal moisturizer, each getting a 6-month supply. Active Group-H: received an over-the-counter product containing a form of hyaluronic acid. Active Group-P: received an over-the-counter moisturizer labeled as prebiotic to promote healthy lactobacilli. Application of vaginal moisturizer was performed daily during Week 1, and then 2–3 times weekly thereafter. Provision of water-based lubricants and/or silicone vaginal dilator for application during sexual activity. Control: Usual Care	Sexual function Distress about sexual problems Dyspareunia Data collected at: Baseline 6 months after randomization (T1) 12 months after randomization (T2)	Female Sexual Function Index (FSFI) Menopausal Sexual Interest Questionnaire Female Sexual Distress Scale-Revised Breast Cancer Prevention Trial Symptom Scale
Nyrop et al., 2017 [35]; USA	Participants’ home	Breast cancer survivors previously diagnosed with stage 0–III cancer, undergoing aromatase inhibitor therapy *N* = 62 Intervention: 31 Control: 31 Duration of adjuvant endocrine therapy undergone by subjects: All participants: 1.7 ± 1.43 years (not specified for each group)	Physical activity intervention	Home-based walking program Intervention: Participation in a 6-week walking program where participants were encouraged to reach the target of having walked 150 min per week. Provision of a brochure with topics on the importance of physical activities (walking) and symptoms associated with cancer treatment (joint pain). Provision of activity log to record physical activity level daily. Control: Wait-list control, receiving intervention after post-intervention data collection	Joint symptoms QOL Pain Fatigue Data collected at: Baseline Immediately post-intervention (T1)—6 weeks follow-up 6 months post-intervention (T2)	Visual Analog Scale (VAS) Western Ontario and McMaster Universities Arthritis Index Functional Assessment of Cancer Therapy-General (FACT-G)
Rogers et al., 2017 [48]; USA	Local institutions, with home-based exercises	Breast cancer survivors (stages I–IIIA), receiving hormonal therapy *N* = 222 Intervention: 110 Control: 112 Duration of adjuvant endocrine therapy undergone by subjects: Not specified	Physical activity intervention	Physical activity behavior change intervention (Better Exercise Adherence after Treatment for Cancer) Intervention: Attendance to 12 supervised exercise sessions for 6 weeks, supplemented by unsupervised home-based exercises. Attendance to counselling sessions with exercise specialists every 2 weeks. Attendance to 6 group discussion sessions on topics including the benefits of and barriers to doing exercises, goal setting for exercise levels. Control: Usual care	Sleep quality Data collected at: Baseline Immediately post-intervention (T1) 3 months post-intervention (T2)	Pittsburgh Sleep Quality Index
Hershman et al., 2015 [39]; USA	Not specified	Breast cancer survivors (stages I–III), receiving adjuvant aromatase inhibitor therapy *N* = 262 Intervention: 131 Control: 131 Duration of adjuvant endocrine therapy undergone by subjects: Intervention group: 1.1 years (median) Control group: 1.3 years (median)	Dietary intervention	Dietary intervention with omega-3 fatty acids Intervention: Intake of 6 capsules containing 3.3 g omega-3 fatty acid (eicosapentaenoic acid and docosahexaenoic acid) per day, over a period of 24 weeks. Control: Intake of placebo capsule containing soybean oil and corn oil, 6 per day over 24 weeks.	Joint symptoms (pain and stiffness) Functional status Data collected at: Baseline Week 6 of intervention Week 12 of intervention Week 24 of intervention (immediately post-intervention)	Brief Pain Inventory (BPI) Western Ontario and McMaster Universities Osteoarthritis Index (WOMAC) Modified Score for the Assessment and Quantification of Chronic Rheumatoid Affections of the Hands (MSACRAH) Serum C-reactive protein (CRP) levels (objective measure of pain)
Irwin et al., 2015 [38], USA	Local health club and home-based	Breast cancer Survivors (stages I–III), undergoing aromatase inhibitor therapy *N* = 121 Intervention group = 61 Control Group = 60 Duration of adjuvant endocrine therapy undergone by subjects: Intervention group: 1.9 ± 2.9 years Control group: 1.8 ± 1.3 years	Physical activity intervention	Year-long exercise intervention Intervention Group: Participation in a supervised resistance exercise program with sessions held twice a week, and a home-based aerobic exercise program (brisk walking or stationary cycling) of 150 min per week. The program lasted for 1 year. Control Group Usual care Participants in both groups were given education booklets with information on breast cancer-related symptoms including lymphedema and fatigue	Joint pain Data collected at Baseline 3 months after randomization (T1) 6 months after randomization (T2) 9 months after randomization (T3) 12 months after randomization (T4)	Brief Pain Inventory Western Ontario and McMaster Universities Osteoarthritis Index (WOMAC)
Peppone et al., 2015 [45]; USA	Community-based and group-based	Breast cancer survivors (stages 0–III) receiving either tamoxifen or aromatase inhibitor therapy *N* = 167 Intervention: 75 Control: 92 Duration of adjuvant endocrine therapy undergone by subjects: Not specified	Psychotherapeutic intervention	Yoga intervention Intervention: Attendance to group sessions of yoga, involving breathing and mindfulness exercises and physical alignment postures. Each session lasted 75 min, and was held twice a week over a period of 4 weeks. Control: Wait-list control, receiving standard care during the intervention period.	Fatigue Data collected at: Baseline Immediately post-intervention	Selected items from: University of Rochester Cancer Center Symptom Inventory Functional Assessment of Chronic Illness Therapy with Fatigue Subscale (FACIT-F) Multidimensional Fatigue Symptom Inventory-Short Form (MFSI-SF)
Mao et al., 2014 [46], USA	Tertiary care academic medical center	Breast cancer patients (stages I–III), currently undergoing aromatase inhibitor therapy *N* = 67 Intervention = 22 Sham acupuncture control group = 22 Waitlist Control = 23 Duration of adjuvant endocrine therapy undergone by subjects: Intervention group: 26.9 ± 17.3 months Sham acupuncture control group: 19.5 ± 16.9 months Control group: 31.1 ± 22.1 months	Acupuncture intervention	Electroacupuncture (EA) intervention Intervention Undertake 30-min electroacupuncture sessions, held twice a week for 2 weeks, then weekly for 6 more weeks Sham Electroacupuncture (SA) Undertake 30-min electroacupuncture sessions, held twice a week for 2 weeks, then weekly for 6 more weeks, but with non-penetrating needles. Waitlist Control Usual care	Fatigue Sleep Anxiety Depression Data collected at: Baseline Week 2 of intervention (T1) Week 4 of intervention (T2) Week 8 of intervention (T3) 4 weeks post-intervention (T4)	Brief Fatigue Inventory (BFI) Pittsburgh Sleep Quality Index (PSQI) Hospital Anxiety and Depression Scale (HADS)
Oh et al., 2013 [41], Australia	Tertiary Teaching Hospital	Breast cancer patients (stages I–IIIa), undergoing aromatase inhibitor therapy *N* = 29 Intervention group = 14 Sham Electroacupuncture control group = 15 Duration of adjuvant endocrine therapy undergone by subjects: All participants: At least 6 months	Acupuncture intervention	Electroacupuncture (EA) intervention Intervention Group: Undertake 20-min sessions of real electroacupuncture twice weekly for 6 weeks, using acupuncture needles Control Group: Undertake 20-min sessions of sham electroacupuncture twice weekly for 6 weeks, using sham acupuncture needles that do not penetrate the skin	Joint pain and joint stiffness QOL Data collected at: Baseline Immediate post-intervention (T1) 6-months post-intervention (T2)	Brief Pain Inventory Short Form Western Ontario and McMaster Universities Osteoarthritis Index Functional assessment of Cancer Therapy-General Symptoms
Mann et al., 2012 [30]; United Kingdom	Not specified	Breast cancer survivors (stage unknown), undergoing endocrine therapy *N* = 96 Intervention: 47 Control: 49 Duration of adjuvant endocrine therapy undergone by subjects: Not specified	Psychotherapeutic intervention	Cognitive behavioral therapy (CBT) Intervention: Attendance to six 90-min group-based CBT sessions, held once a week, over a period of 6 weeks. These sessions included the provision of psycho-education via presentations and handouts, group discussions and homework assignments. Control: Usual care	Perceived burden of hot flush and night sweats Emotional symptoms Sleep problems HRQOL Data collected at: Baseline 9 weeks after randomization (T1) 26 weeks after randomization (T2)	Hot Flush Rating Scale Women’s Health Questionnaire General Health Survey Short Form 36
Zhao et al., 2012 [44]; China	Not specified	Breast cancer survivors previously diagnosed with stage I–IIIA cancer, having completed or undergoing endocrine therapy *N* = 48 Intervention: 25 Control: 23 Duration of adjuvant endocrine therapy undergone by subjects: All participants: 6 months–5 years (not specified for each group)	Pharmacological intervention	Pharmacological intervention with spore powder of G. lucidum Intervention: Intake of 1000 mg spore powder of *G. lucidum* three times a day for 4 weeks Control: Intake of placebo, three times a day for 4 weeks	Fatigue Anxiety and depression QOL Data collected at: Baseline Post-intervention	Functional Assessment of Cancer Therapy: Fatigue (FACT-F) The Hospital Anxiety and Depression Scale European Organization for Research and Treatment of Cancer Core Quality of Life Questionnaire C30 (EORTC-CLC-C30) Seral level of TNF-α and IL-6 (objective measurement of fatigue)
Rogers et al., 2009 [36]; USA	Not specified	Breast cancer survivors (stages I–IIIA), currently on aromatase inhibitor therapy or estrogen receptor modulator therapy *N* = 41 Intervention: 21 Control: 20 Duration of adjuvant endocrine therapy undergone by subjects: All participants: 18 ± 17 months (not specified for each group)	Physical activity intervention	Physical activity behavior change intervention (The BEAT Cancer Program) Intervention: During the 12-week intervention, participants attended 12 individual supervised exercise sessions for the first 6 weeks and 3 counselling sessions face-to-face with an exercise specialist for the next 6 weeks to tailor a home-based exercise program at the end of the intervention and enhance the ability of participants to self-monitor their physical activity level Participation in 6 group discussion sessions focusing on journaling, time and stress management, barriers to exercise and behavioral change Control: Usual care	QOL Sleep quality Joint symptoms Data collected at: Baseline Immediately post-intervention (T1) 3 months post-intervention (T2)	Functional Assessment of Cancer Therapy-Breast (FACT-B) Pittsburgh Sleep Quality Index Western Ontario and McMaster Universities Osteoarthritis Index
Rogers et al., 2009 [37]; USA	Not specified	Same as Rogers et al, 2009 [36]	Physical activity intervention	Same as Rogers et al., 2009 [36]	QOL Sleep quality Joint symptoms Data collected at: Baseline Immediately post-intervention	Functional Assessment of Cancer Therapy-Breast (FACT-B) Pittsburgh Sleep Quality Index (PSQI) Western Ontario and McMaster Universities Osteoarthritis Index
Crew et al., 2007 [42]; USA	Not specified	Breast cancer survivors (stages I–IIIa), undertaking aromatase inhibitor therapy *N* = 21 Intervention: Not mentioned Control: Not mentioned Duration of adjuvant endocrine therapy undergone by subjects: All participants: At least 6 months (not specified for each group)	Acupuncture intervention	Acupuncture intervention Intervention: Acupuncture session lasting 30 min, performed two times a week for six weeks. Control: Wait-list control	Joint pain and stiffness QOL Serum levels of inflammatory markers, IL-1β and TNF-α Data collected at: Baseline 6 weeks after randomization (T1) 12 weeks after randomization (T2)	Brief Pain Inventory-Short Form (BPI-SF) Western Ontario and McMaster Universities Osteoarthritis (WOMAC) index The Functional Assessment of Cancer Therapy-General quality of life measure Enzyme-linked immunosorbent assay (ELISA)
Kimmick et al., 2006 [27]; USA	Not specified	Breast cancer survivors (stages 0–IIIB), receiving tamoxifen therapy *N* = 62 Intervention: 33 Control: 29 Duration of adjuvant endocrine therapy undergone by subjects: Not specified	Pharmacological intervention (a crossover trial)	Pharmacological intervention with the antidepressant sertraline Intervention: Intake of 50 mg sertraline per day, over a period of 6 weeks. Intervention participants then took the placebo tablets per day for the next 6 weeks. Control: Intake of placebo tablet per day, over a period of 6 weeks. Control participants then took 50 mg sertraline per day for the next 6 weeks.	Hot flashes frequency and severity Depression QOL Data collected at: Baseline 6 weeks after start of intervention (T1) 12 weeks after start of intervention (T2)	Daily diary for hot flashes Center for Epidemiologic Studies depression (CESD) Functional Assessment of Cancer Therapy—Breast (FACT-B)
Pandya et al., 2000 [28]; USA	University of Rochester Cancer Centre	Breast cancer survivors (stage unknown), receiving adjuvant tamoxifen therapy *N* = 198 Intervention: 99 Control: 99 Duration of adjuvant endocrine therapy undergone by subjects: All participants: At 1 month (not specified for each group)	Pharmacological intervention	Pharmacological intervention with clonidine Intervention: Intake of 0.1 mg oral clonidine, once daily, for a period of 8 weeks. Control: Intake of 0.1 mg placebo, once daily, for a period of 8 weeks.	Hot flashes duration, frequency and severity QOL Data collected at: Baseline 4 weeks post-randomization (T1) 8 weeks post-randomization (T2) 12 weeks post-randomization (T3)	Daily diary for hot flashes A 10-point rating scale (for QOL assessment)
Goldberg et al., 1994 [29]; USA	Not specified	Breast cancer patients (stage unknown), receiving tamoxifen therapy *N* = 110 Intervention: 55 Control: 55 Duration of adjuvant endocrine therapy undergone by subjects: Not specified	Pharmacological intervention	Pharmacological intervention with transdermal clonidine Intervention: Administered transdermal clonidine patch (equivalent to the oral dose of 0.1 mg of the drug) daily for a period of 4 weeks, then intervention participants were administered the placebo transdermally daily for the next 4 weeks. Control: Administered the placebo patch to be used daily for a period of 4 weeks, then control participants were administered the transdermal clonidine patch (equivalent to the oral dose of 0.1 mg of the drug), used daily for the next 4 weeks.	Hot flashes frequency and severity Data collected at: Baseline Immediately post-intervention Post-crossover	Daily patient questionnaire

Abbreviations: QOL, quality of life; HRQOL, health-related quality of life.

**Table 4:** A summary of the effects of reported interventions on individual adjuvant endocrine therapy-induced menopausal symptoms and sexual issues.

Menopausal Symptom/Issue	Intervention Type	Intervention Name	Major Findings on Intervention Effects on Symptom *	Reference
Hot flashes	Pharmacological intervention	Pharmacological intervention with tablets of a medicinal plant extract (*Paullinia cupana* or Guarana)	No significant difference in the extent of decrease in frequency (*p* = 0.54) and intensity (*p* = 0.84) of hot flashes between groups. Both groups exhibited a decrease in these parameters.	de Sousa Vieira et al., 2019 [25]
		Pharmacological intervention with a homeopathic medicine named Actheane^®^	(T1: 4 weeks after randomization; T2: 8 weeks after randomization) There was no difference in the hot flashes score between the intervention and control participants at T1 (*p* = 0.756), and T2 (*p* = 0.775). Both groups exhibited a decrease in hot flashes score between baseline and T1/T2. No significant differences in mean daily hot flashes frequency or intensity between groups at all data collection time points (*p* values not reported). However, at both T1 and T2, the majority of the participants exhibited a decrease in mean daily hot flashes frequency (71% and 74%, respectively), while some of them had decreased mean daily hot flashes intensity (21% and 27%, respectively). Almost half of the participants expressed that the impact of hot flashes on their quality of life has been reduced at both T1 (47%) and T2 (50%).	Heudel et al., 2019 [26]
		Pharmacological intervention with the antidepressant sertraline	Before cross-over at 6 weeks after start of intervention (T1) No significant between-group differences in the hot flashes frequency (*p* = 0.80) and hot flashes score (*p* = 0.50). No significant difference in the proportion of participants achieving 50% reduction of hot flashes frequency between intervention and control groups (36% vs. 27%; *p* = 0.70). After cross-over at 12 weeks after start of intervention (T2) Significant between-group difference was observed in terms of hot flashes frequency (*p* = 0.03) and hot flashes score (*p* = 0.03). For both parameters, differential outcome was observed, where an improvement in both parameters was observed in control group, and worsening was observed in intervention group.	Kimmick et al., 2006 [27]
		Pharmacological intervention with clonidine	(T1: 4 weeks after randomization; T2: 8 weeks after randomization; T3: 12 weeks after randomization) Intervention participants exhibited a significantly greater reduction in the number of daily hot flashes (*p* ≤ 0.006) and hot flashes scores (*p* ≤ 0.006) at T1 and T2, compared to controls. However, difference in the extent of reduction between the two groups was not significant for both parameters at T3. Intervention participants had a significantly greater reduction in hot flashes duration only at T3 (*p* = 0.023), but not at T1 (*p* = 0.11) or T2 (*p* = 0.18). No significant difference in extent of reduction in hot flashes severity between groups at all time points of outcome assessment (*p* ≥ 0.08).	Pandya et al., 2000 [28]
		Pharmacological intervention with transdermal clonidine	Intervention participants exhibited a significantly greater extent of reduction in hot flashes frequency (20% more than controls; *p* < 0.0001), hot flashes severity (10% more than controls; *p* = 0.02) and hot flashes score (27% more than controls; *p* = 0.0006) as a result of the use of transdermal clonidine.	Goldberg et al., 1994 [29]
	Psychotherapeutic intervention	Cognitive behavioral therapy	(T1: 9 weeks after randomization; T2: 26 weeks after randomization) The intervention participants exhibited a significantly greater decrease in the level of perceived burden of hot flashes compared to controls, at both T1 and T2 (*p* < 0.0001). No significant between-group differences were observed in the level of reduction in hot flashes frequency, at both T1 and T2.	Mann et al., 2012 [30]
Night sweats	Psychotherapeutic intervention	Cognitive behavioral therapy	(T1: 9 weeks after randomization; T2: 26 weeks after randomization) The intervention participants exhibited a significantly greater decrease in the level of perceived burden of night sweats compared to controls, at both T1 and T2 (*p* < 0.0001). No significant between-group differences were observed in the level of reduction in night sweats frequency, at both T1 and T2.	Mann et al., 2012 [30]
Vaginal atrophy	Miscellaneous intervention	Vitamin D/vitamin E vaginal suppository intervention	(T1: 2 weeks after start of intervention; T2: 4 weeks after start of intervention; T3: 8 weeks after start of intervention) Within-group comparison Significant decreases in the mean score of the genitourinary atrophy self-assessment were observed among the participants in the vitamin D and vitamin E groups over the 8-week intervention (*p* < 0.001). No significant difference was observed in this score among the participants in the placebo group (*p* = 0.564). Between-group comparison The mean score of the genitourinary atrophy self-assessment was significantly lower among the participants in the vitamin D and vitamin E groups compared to controls, at T1 (*p* = 0.017), T2 (*p* < 0.001) and T3 (*p* < 0.001).	Keshavarzi et al., 2019 [31]
Sexual issues	Pharmacological intervention	Pharmacological intervention with tablets of a medicinal plant extract (*Paullinia cupana* or Guarana)	No significant difference was observed between groups (*p* = 0.60).	de Sousa Vieira et al., 2019 [25]
	Physical activity intervention	Supervised combined exercise training intervention	No significant time × group interaction for sexual functioning (*p* = 0.77) or sexual enjoyment (*p* = 0.16) scores.	Paulo et al., 2019 [32]
	Multimodal intervention	A multimodal intervention involving sexual counseling, and use of vaginal moisturizers, lubricants, and/or dilator	(T1: 6 months after randomization; T2: 12 months after randomization) No significant differences in % of women with sexual dysfunction between treatment groups at baseline, T1 and T2 any of the three time points of data collection. At T1, Active Group-H exhibited more significant improvement in sexual function compared with Active Group-P, in terms of the FSFI total score (*p* = 0.04). At T1, the control participants had more dyspareunia than the intervention participants did (*p* = 0.07).	Advani et al., 2017 [33]

* Major findings are reported in the form of between-group comparisons unless otherwise state.

**Table 5:** A summary of the effects of reported interventions on various endocrine therapy-induced joint symptoms.

Intervention Type	Intervention Name	Major Findings on Intervention Effects on Symptom *	Reference
Pharmacological intervention	Pharmacological intervention with Duloxetine	(T1: 2 weeks after randomization; T2: 6 weeks after randomization; T3:12 weeks after randomization; T4: 24 weeks after randomization) More significant decrease (0.82 points more) in average pain score was observed among intervention participants compared to controls (*p* = 0.0002). However, by T4, no significant difference in this parameter was observed between groups (*p* = 0.80). Significantly more intervention participants exhibited clinically meaningful improvement in pain at T2, compared to controls (68% vs. 49%, *p* = 0.003). No significant difference between groups for this parameter at other time points. There was a significant improvement of joint pain in knees and hips among intervention participants, compared to controls (*p* < 0.001). At T1, T2 and T3, participants in intervention group showed significantly lower levels of worst joint pain, pain interference and joint stiffness as measured by BPI and GRCS. No significant between-group difference was observed in T4.	Henry et al., 2018 [34]
Physical activity intervention	Home-based walking program	(T1: immediate post-intervention; T2: 6 months post-intervention) Within-group comparisons Intervention group At T1, intervention participants experienced a decrease in joint pain, but the decrease is not statistically significant (*p* value not reported). Nevertheless, participants’ perceived level of joint stiffness (*p* < 0.05) and perceived difficulty with daily activities (*p* < 0.01) decreased significantly after the intervention. By T2, the level of joint pain and stiffness and perceived difficulty with daily activities were still lower compared to baseline, but the difference was non-significant (*p* value not reported). Control group At immediately post-intervention, no significant differences were observed in all the above outcomes when compared to baseline. Between-group comparisons At T1, intervention participants had reduced stiffness scores (*p* < 0.05) and less difficulty with activities of daily living (*p* < 0.01) No significant between-group difference was observed for joint pain and stiffness between T1 and T2 (*p* value not reported).	Nyrop et al., 2017 [35]
	Physical activity behavior change intervention (The BEAT Cancer Program)—pilot study	(T1: immediate post-intervention; T2: 3 months post-intervention) Between baseline and T2, no significant between-group difference was observed for the changes in level of joint pain (*p* = 0.32), joint stiffness (*p* = 0.40) or physical dysfunction of joints (*p* = 0.09). Between baseline and T1, no significant between-group difference on joint pain (*p* = 0.40) or physical dysfunction of joints (*p* = 0.41), but more significant improvement on joint stiffness was observed among intervention participants (*p* = 0.04).	Rogers et al., 2009 [36] Rogers et al., 2009 [37]
	Year-long exercise intervention	(T1: 3 months after randomization; T2: 6 months after randomization; T3: 9 months after randomization; T4: 12 months after randomization) Worst joint pain score was decreased by 29% among intervention participants, while increased by 3% among control participants at T4. (*p* < 0.001). Statistically significant difference was also observed in joint pain severity between intervention and control participants. (*p* < 0.001). WOMAC total score (measure of joint symptoms in lower limbs) was decreased by 37% among intervention participants, while increased by 2% among control participants at T4 (*p* < 0.001).	Irwin et al., 2015 [38]
Dietary intervention	Dietary intervention with omega-3 fatty acids	(T1: Week 6 of intervention; T2: Week 12 of intervention; T3: Week 24 of intervention/immediate post-intervention) Within-group comparisons There was significant reduction in pain from baseline for both intervention and control groups at T1, T2 and T3 (*p* < 0.01). Between-group comparisons There was no significant difference in the worst pain score assessed by BPI between intervention and control groups at all of the time points of measurement (*p* ≥ 0.34). Similar observations were obtained for level of interference on daily activities by pain (*p* ≥ 0.58), global rating in change in joint pain (*p* ≥ 0.16) and joint stiffness pain (*p* ≥ 0.12). There were no significant differences in the perceived joint pain levels (as measured by WOMAC and MSACRAH) (*p* ≥ 0.41) and serum CRP levels (objective measure of joint pain, *p* = 0.71) at all the time points of measurement between intervention and control groups.	Hershman et al., 2015 [39]
Acupuncture intervention	Acupuncture intervention	(T1: 6 weeks after randomization; T2: 12 weeks after randomization) Worst joint pain score The worst joint pain score among the intervention participants was significantly lower than that among participants in both control groups at T1 (lower by 0.92–0.96 points, *p* = 0.01). However, the between-group difference in this outcome was no longer significant at T2 when comparing between intervention group and sham acupuncture control group (*p* = 0.08). Average joint pain The average pain score among the intervention participants was significantly lower than that among participants in both control groups at both T1 (lower by 0.60–0.71 points, *p* ≤ 0.04) and T2 (lower by 0.79–1.38 points, *p* ≤ 0.02). Joint pain severity The pain severity score among the intervention participants was significantly lower than that among participants in both control groups at T1 (lower by 0.56-0.71 points, *p* ≤ 0.05). However, at T2, no significant difference in this parameter was observed between intervention group and sham acupuncture control group (lower by 0.53 points, *p* = 0.08). Worst joint stiffness score The worst joint stiffness score among the intervention participants was significantly lower than that among participants in both control groups at T1 (lower by 1.00–1.09 points, *p* ≤ 0.02). However, at T2, no significant difference in this parameter was observed between intervention group and sham acupuncture control group (lower by 0.72 points, *p* = 0.08).	Hershman et al, 2018 [40]
	Electro-acupuncture intervention	(T1: Immediate post-intervention; T2: 6 months post-intervention) No significant between-group differences were observed in joint pain and joint stiffness at both T1 and T2 (*p* values not reported). However, there was a trend of higher level of improvement in joint stiffness and physical functioning at T2 for the intervention participants, compared with controls.	Oh et al., 2013 [41]
	Acupuncture intervention	Within-group comparisons As measured by BPI-SF for joint pain measurement, significant decrease in worst pain score (*p* = 0.008), pain severity (*p* = 0.022) and pain-related interference (*p* = 0.015) was reported among the intervention participants after receiving acupuncture. As measured by WOMAC, improvement of joint pain (*p* = 0.145) and joint stiffness (*p* = 0.067) was observed among intervention participants after receiving acupuncture, but the level of improvement did not reach statistical significance. Between-group comparisons The opposite effect was observed in the changes of pain severity, joint pain and stiffness between the intervention and control participants (*p* *values not reported).* Despite the improvement of symptoms among intervention participants, such improvement did not persist 6 weeks after the intervention.	Crew et al., 2007 [42]
Miscellaneous intervention	Whole body vibration intervention	No significant differences in the joint pain levels (*p* = 0.334), joint stiffness levels (*p* = 0.224) or level of physical dysfunction of joints (*p* = 0.063) of participants between groups at post-intervention.	Baker et al., 2018 [43]

* Major findings are reported in the form of between-group comparisons unless otherwise stated.

**Table 6:** A summary of the effects of reported interventions on endocrine therapy-induced fatigue.

Intervention Type	Intervention Name	Major Findings on Intervention Effects on Fatigue *	Reference
Pharmacological intervention	Pharmacological intervention with spore powder of *G. lucidum*	Compared to control participants, intervention participants had a more significant improvement FACT-F score (*p* < 0.01).	Zhao et al., 2012 [44]
Physical activity intervention	Supervised combined exercise training intervention	There was a significant time × group interaction for the perceived severity of fatigue among the intervention participants, compared to controls. (*p* = 0.001).	Paulo et al., 2019 [32]
	Home-based walking program	(T1: immediate post-intervention; T2: 6 months post-intervention) Within-group comparisons Both groups exhibited no significant changes on fatigue level as measured by VAS at T1, when compared to baseline. Between-group comparisons There was no significant between-group difference on fatigue level between T1 and T2 (*P* values not reported).	Nyrop et al., 2017 [35]
Psychotherapeutic intervention	Yoga intervention	Intervention participants also perceived a significantly greater reduction in fatigue levels indicated by the FACIT-F physical subscale score and MFSI-SF physical subscale score at post-intervention (both *p* = 0.001).	Peppone et al., 2015 [45]
Acupuncture intervention	Electro-acupuncture intervention	(T1: Week 2 of intervention; T2: Week 4 of intervention; T3: Week 8 of intervention/immediate post-intervention; T4: 4 weeks post-intervention) Intervention participants showed more significant improvement in fatigue over time compared with wait-list control participants (*p* = 0.0095). Greater reduction in BFI score (measure of fatigue) was observed among intervention participants compared to wait-list controls at T3 (*p* = 0.0034), and this reduction effect persisted at T4 (*p* = 0.022).	Mao et al., 2014 [46]
Miscellaneous intervention	Whole body vibration intervention	There were no significant differences in the perceived fatigue levels (*p* = 0.079) of participants between groups at post-intervention.	Baker et al., 2018 [43]
	Neuromuscular taping intervention	(T1: 1 week after start of intervention/immediate post-intervention; T2: 5 weeks after start of intervention/4 weeks post-intervention) Within-group comparisons Intervention group Significant improvement was observed for fatigue (*p* = 0.00) between baseline, T1 and T2. Control group No significant improvement was observed for fatigue (*p* ≥ 0.46) between baseline, T1 and T2. Between-group comparisons There was no significant between-group difference in fatigue score at T1 (*p* = 0.07) However, by T2, intervention group had significantly higher score for fatigue (*p* = 0.01) compared to controls.	Conejo et al., 2018 [47]

* Major findings are reported in the form of between-group comparisons unless otherwise stated.

**Table 7:** A summary of the effects of reported interventions on endocrine therapy-induced sleep disturbance.

Intervention Type	Intervention Name	Major Findings on Intervention Effects on Sleep Disturbance *	Reference
Physical activity intervention	Supervised combined exercise training intervention	A significant time × group interaction for the perceived severity of sleep. (*p* = 0.04)	Paulo et al., 2019 [32]
	Physical activity behavior change intervention (The BEAT Cancer Program)	(T1: immediate post-intervention; T2: 3 months post-intervention) Within-group comparisons Intervention group Significantly improved sleep quality was observed among intervention participants at both T1 and T2, when compared to that at baseline. Between-group comparisons Intervention participants showed a more significant improvement in sleep quality (*p* = 0.002) and level of sleep disturbance (*p* = 0.016) at T1, when compared to control participants. Nevertheless, differences in the extent of improvement in both parameters were no longer significant between groups at T2 (*p* = 0.41 for sleep quality and *p* = 0.11 for sleep disturbance).	Rogers et al., 2017 [48]
	Physical activity behavior change intervention (The BEAT Cancer Program)—pilot study	(T1: immediate post-intervention; T2: 3 months post-intervention) Significant group effect was observed at T2 for sleep latency (*p* = 0.048), but not for other sleep parameters (habitual sleep efficiency, sleep duration, subjective sleep quality, use of sleeping medication and daytime dysfunction). There was no significant between-group difference at T1 on outcomes for sleep (sleep quality, sleep latency, sleep duration, sleep efficiency, sleep medications and daytime dysfunction) as well as total PSQI score (*p* ≥ 0.08).	Rogers et al., 2009 [36] Rogers et al., 2009 [37]
Psychotherapeutic intervention	Cognitive behavioral therapy	(T1: 9 weeks after randomization; T2: 26 weeks after randomization) A significantly greater alleviation in sleep difficulties was observed among intervention participants compared to controls at both T1 (*p* < 0.0001) and T2 (*p* < 0.05).	Mann et al., 2012 [30]
Acupuncture intervention	Electro-acupuncture intervention	(T1: Week 2 of intervention; T2: Week 4 of intervention; T3: Week 8 of intervention/immediate post-intervention; T4: 4 weeks post-intervention) There were no significant differences in the extent of improvement over time on PSQI score (measure of sleep quality) between intervention and wait-list control group (*p* = 0.058). No significant improvement was observed among intervention participants in the PSQI score at T3 (*p* = 0.087).	Mao et al., 2014 [46]
Miscellaneous intervention	Neuromuscular taping intervention	(T1: 1 week after start of intervention/immediate post-intervention; T2: 5 weeks after start of intervention/4 weeks post-intervention) Within-group comparisons Intervention group Significant improvement was observed for insomnia (*p* ≤ 0.009) between baseline, T1 and T2. Control group Significant improvement was also observed for insomnia (*p* ≤ 0.02) between baseline, T1 and T2. Between-group comparisons No significant between-group differences were observed (*p* = 1.00) for insomnia at T1 and T2.	Conejo et al., 2018 [47]

* Major findings are reported in the form of between-group comparisons unless otherwise stated.

**Table 8:** A summary of the effects of reported interventions on participants’ QOL and functional ability.

Outcome	Intervention Type	Intervention Name	Major Findings on Intervention Effects on QOL/Functional Ability *	Reference
QOL	Pharmacological intervention	Pharmacological intervention with tablets of a medicinal plant extract (*Paullinia cupana* or Guarana)	No significant difference was observed between groups on participants’ QOL (*p* value not reported).	de Sousa Vieira et al., 2019 [25]
		Pharmacological intervention with Duloxetine	There was significant improvement of functional QOL among intervention participants, compared to controls (*p* = 0.009).	Henry et al., 2018 [34]
		Pharmacological intervention with spore powder of *G. lucidum*	There was a more significant improvement in scores of various QOL domains, including emotional functioning (*p* < 0.05), cognitive functioning (*p* < 0.05), physical functioning (*p* < 0.01) and global QOL (*p* < 0.01).	Zhao et al., 2012 [44]
		Pharmacological intervention with the antidepressant sertraline	(T1: Before cross-over at 6 weeks after start of intervention; T2: After cross-over at 12 weeks after start of intervention) No significant difference was observed in FACT-B score between groups at both before cross-over at T1 (*p* = 0.32) and after cross-over at T2 (*p* = 0.88).	Kimmick et al., 2006 [27]
		Pharmacological intervention with clonidine	(T1: 4 weeks after randomization; T2: 8 weeks after randomization; T3: 12 weeks after randomization) Compared to controls, intervention participants exhibited a significantly greater improvement QOL score at T1 (*p* = 0.003) and T2 (*p* = 0.022), but the difference in the extent of such improvement between groups was not significant at T3 (*p* > 0.20).	Pandya et al., 2000 [28]
	Physical activity intervention	Supervised combined exercise training intervention	(T1: 12 weeks after start of intervention; T2: 24 weeks after start of intervention; T3: 36 weeks after start of intervention) A significant time × group interaction was observed for the scores for role functioning domain in EORTC-CLC-C30 (*p* = 0.01), and most of the domains in SF-36 (*p* ≤ 0.02) at T2 and T3.	Paulo et al., 2019 [32]
		Home-based walking program	(T1: immediate post-intervention; T2: 6 weeks post-intervention) Within-group comparisons Intervention group No significant changes were observed among intervention participants the score for emotional well-being and functional well-being (as measured by FACT-G) at T1, when compared to baseline. Control group At T1, none of the FACT-G domains exhibited significant differences when compared to baseline. Between-group comparisons There was no significant between-group difference on the scores of all FACT-G domains between T1 and T2.	Nyrop et al., 2017 [35]
		Physical activity behavior change intervention (The BEAT Cancer Program)—pilot study	(T1: immediate post-intervention; T2: 3 months post-intervention) Between baseline and T1, no significant between-group differences were observed in overall QOL or any sub-scales of FACT-B, except a significantly greater extent of improvement on social well-being (*p* = 0.03). Between baseline and T2, a significantly greater extent of improvement on social well-being (*p* = 0.03) and overall QOL (*p* = 0.045) was noted for intervention group. However, none of the other subscales of FACT-B (physical well-being, emotional well-being, functional well-being and additional concerns) showed significant between-group differences.	Rogers et al., 2009 [36] Rogers et al., 2009 [37]
	Psychotherapeutic intervention	Cognitive behavioral therapy	(T1: 9 weeks after randomization; T2: 26 weeks after randomization) A more significant improvement on general health was observed at both T1 (*p* < 0.05) and T2 (*p* < 0.01) among intervention participants compared to controls. A more significant improvement on HRQOL domains including physical functioning (*p* < 0.05) and social functioning (*p* < 0.01) was observed among intervention participants compared to controls, but these improvements were only observed at T2.	Mann et al., 2012 [30]
	Acupuncture intervention	Electro-acupuncture intervention	(T1: Immediate post-intervention; T2: 6 months post-intervention) No significant between-group differences were observed in QOL at both T1 and T2 (*p* values not reported). However, there was a trend of higher level of improvement in physical functioning at T2 for the intervention participants, compared with controls.	Oh et al., 2013 [41]
		Acupuncture intervention	Within-group comparison Intervention participants experienced a significant improvement in physical well-being after receiving acupuncture (*p* = 0.03), but no significant changes were observed for functional, social and emotional well-being (*p* ≥ 0.127). Between-group comparisons Compared to controls, intervention participants exhibited more significant improvement on their QOL after receiving the intervention (*p* values not reported). Such improvement did not persist 6 weeks after the intervention.	Crew et al., 2007 [42]
	Miscellaneous intervention	Neuromuscular taping intervention	(T1: 1 week after start of intervention/immediate post-intervention; T2: 5 weeks after start of intervention/4 weeks post-intervention) Within-group comparisons Intervention group Significant improvement was observed in global health status (*p* = 0.002), role functioning (*p* = 0.03) and emotional functioning (*p* = 0.008) between baseline and T1. By T2, significant improvement was observed among all functional scales in the EORTC-CLC-C30 (*p* < 0.02) and global health status (*p* = 0.000). Control group No significant improvement was observed for all QOL domains at T1 and T2 (*p* ≥ 0.41). Between-group comparisons No significant differences were observed in all QOL outcomes between the intervention and control groups at T1 (*p* ≥ 0.06). However, by T2, intervention group had significantly higher score for global health status/QOL (*p* = 0.005), compared to controls.	Conejo et al., 2018 [47]
Functional ability	Physical activity intervention	Home-based walking program	A more significant improvement in the level of difficulties with activities of daily living owing to the joint symptoms was observed among intervention participants, compared to controls (*p* < 0.01).	Nyrop et al., 2017 [35]
	Miscellaneous intervention	Whole body vibration intervention	No significant differences in the changes in functional ability between participants in both groups, as indicated in the results for chair rise (*p* = 0.292) and stair climb (*p* = 0.154) exercises.	Baker et al., 2018 [43]

* Major findings are reported in the form of between-group comparisons unless otherwise stated.
